# Electrocardiogram Changes with Acute Alcohol Intoxication: A Systematic Review

**DOI:** 10.2174/1874192401812010001

**Published:** 2018-02-12

**Authors:** Hitesh Raheja, Vinod Namana, Kirti Chopra, Ankur Sinha, Sushilkumar Satish Gupta, Stephan Kamholz, Norbert Moskovits, Jacob Shani, Gerald Hollander

**Affiliations:** 1 Cleveland Clinic, Ohio, USA; 2 Maimonides Medical Center, Brooklyn, USA; 3 Indiana University School of Dentistry, Indianapolis, USA

**Keywords:** ECG, Alcohol, Alcohol and arrhythmia, Alcohol intoxication, Ethanol toxicity

## Abstract

**Background:**

Acute alcohol intoxication has been associated with cardiac arrhythmias but the electrocardiogram (ECG) changes associated with acute alcohol intoxication are not well defined in the literature.

**Objective:**

Highlight the best evidence regarding the ECG changes associated with acute alcohol intoxication in otherwise healthy patients and the pathophysiology of the changes.

**Methods:**

A literature search was carried out; 4 studies relating to ECG changes with acute alcohol intoxication were included in this review.

**Results:**

Of the total 141 patients included in the review, 90 (63.8%) patients had P-wave prolongation, 80 (56%) patients had QTc prolongation, 19 (13.5%) patients developed T-wave abnormalities, 10 (7%) patients had QRS complex prolongation, 3 (2.12%) patients developed ST-segment depressions.

**Conclusion:**

The most common ECG changes associated with acute alcohol intoxication are (in decreasing order of frequency) P-wave and QTc prolongation, followed by T-wave abnormalities and QRS complex prolongation. Mostly, these changes are completely reversible.

## 1 INTRODUCTION

Environmental factors are important in the pathogenesis of cardiovascular disease. Smoking, diet and physical activity are major ecological factors affecting cardiac health. The effect of alcohol intoxication on the electrocardiogram (ECG) is usually difficult to determine due to absent knowledge of the preexisting condition of the heart [1]. According to the World Health Organization (WHO), alcohol use causes about 2.5 million deaths annually and is the leading risk factor worldwide for deaths among males between 15 and 59 years of age [2]. Acute alcohol intake in otherwise healthy subjects may predispose to cardiac arrhythmias [3]. Chronic heavy alcohol consumption can result in systolic and diastolic dysfunction, left ventricular dilatation, conduction abnormalities, and decreased ejection fraction resulting in alcoholic cardiomyopathy [4, 5]. While light to moderate alcohol consumption (2 drinks/day, 28 g for males and 1 drink, 14 g/day for women) has shown to have cardio-protective benefits, binge drinking has been associated with cardiac dysfunction, stroke, arrhythmias and sudden cardiac death [2]. Up to 15-20% of the patients with acute alcohol intoxication have atrial fibrillation, while others may have different supraventricular and ventricular arrhythmia [6]. On the electrocardiogram (ECG), nonspecific variations from normal may include alterations in the ST segment, P-wave changes, complete or incomplete left bundle branch block or atrioventricular conduction disturbances [7]. We performed a best evidence review of the literature, and in this report, we describe the ECG changes associated with acute alcohol intoxication in otherwise healthy individuals.

## 2 MATERIAL AND METHODS

A PubMed and Cochrane literature search was conducted utilizing Preferred Reporting Items for Systematic Reviews and Meta-Analyses (PRISMA) guidelines. Both structured medical subject headings (MeSH) terms and free terms were used in the search. MeSH words ‘ECG’; ‘alcohol’; ‘ethanol’; ‘acute’ and ‘intoxication’ were used to retrieve literature from the electronic databases. All articles published up to January 2017 were identified. Initially, titles and abstracts were screened. When unclear for inclusion, full-text reports were read to assess eligibility. Reference lists of the selected articles were also checked based on the aforementioned criteria. Duplicates were removed. The literature was selected on the basis of the following criteria:

Inclusion criteria:

1: Randomized controlled trials, case control studies; in English language confirming changes in ECG with acute alcohol intoxication.
2: Studies including human participants of any age and gender with a detectable variation from a normal ECG.

Exclusion criteria:

1: Publications including patients with pre-existing alcohol or non-alcohol related cardiac conditions.
2: Experimental or laboratory studies.
3: Letters, editorials, case reports, doctoral theses and abstracts.

**Fig. (1):**
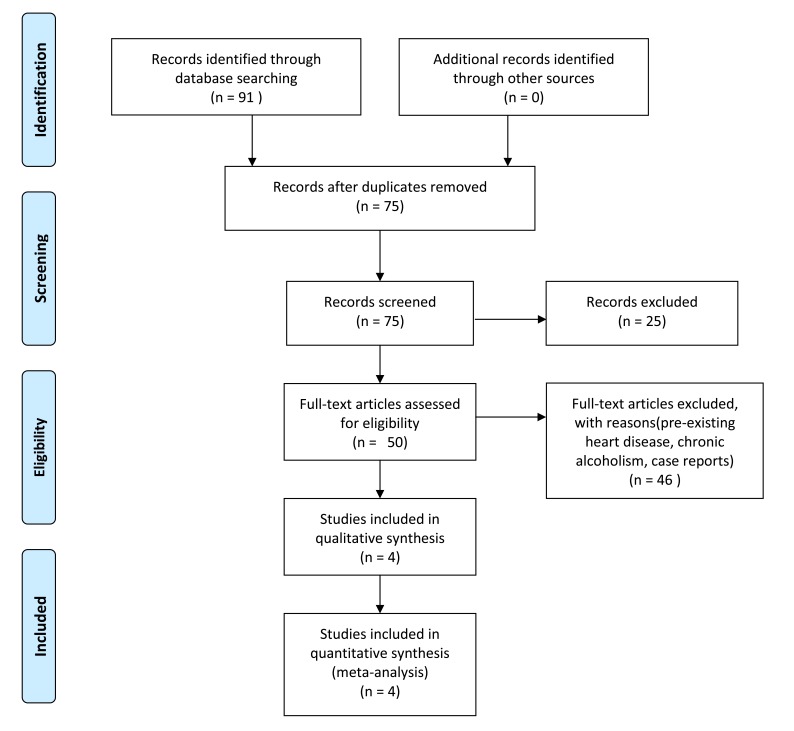
PRISMA flow diagram depicting the selection process of studies.

Ninety-one papers were identified, out of which 4 publications [3, 8-10] (Fig. **1**, Table **1**) met the inclusion criteria. The parameters cataloged from these studies included: 1) Type of study 2) Patient demographics 3) Triggering event 4) Type of ECG changes or arrhythmias 5) Amount of alcohol consumption. We posed the question: “In patients with acute alcohol intoxication, is there a link between alcohol consumption and ECG changes from normal baseline and what is the pathophysiology of the changes?”

## 3 RESULTS

Of the 141 patients included in the review, 90 (63.8%) patients had P-wave prolongation, 80 (56%) patients had QTc prolongation, 19 (13.5%) patients developed T-wave abnormalities, 10 (7%) patients had QRS complex prolongation, 3 (2.12%) patients developed ST-segment depressions and 1 (0.7%) patient was reported as atrial fibrillation (Table **2**).

### 3.1 Changes in P-wave, QRS Complex and QT Interval

Cardy *et al*. [9] reported that alcohol ingestion affects P-wave duration and the QRS complex. The P-wave was prolonged from baseline in 9 of 10 subjects and the QRS complex was prolonged in all subjects, in contrast to the control group. Urayel *et al*. [8] reported P-wave prolongation in 10 subjects with alcohol intoxication compared with the control group, with percentage increase of 9.1% from the baseline. Aaesebo *et al*. [3] also reported that the P-wave and the corrected QT intervals were longer in the ethanol intoxicated group (84 patients) compared with the control group, after adjustment for age, heart rate, comorbidities and serum sodium. P-wave was prolonged in 71 of 84 patients (p=0.09) and QTc was prolonged in 80 patients. There was no difference between the ethanol group and controls in any of the ECG variables at discharge, after adjusting for age, heart rate and comorbidities.

### 3.2 ST Segment and T-wave Changes

Priest *et al*. [10] reported that 20 out of the 37 patients with acute alcohol intoxication had abnormal ECGs. ST-segment depressions were observed in 3 out of 20 patients. They also noted T-wave abnormalities in the majority of their subjects. Of the 20 abnormal ECGs, 19 had abnormal T-waves, 1 had spinous T-wave, 12 had cloven T- waves, 2 had dimple T-waves, 1 had flat isoelectric T-wave and 3 had inverted T-waves. Within several days of admission, most of the cases had disappearance of these T-wave abnormalities.

### 3.3 Atrial Fibrillation

Out of the 4 studies included, Aasebo *et al*. (3) mentioned 1 case of transitory atrial fibrillation.

## 4 DISCUSSION

Due to the absence of other underlying cardiac disorders, arrhythmias occurring in association with acute alcohol intoxication are usually misdiagnosed as idiopathic. Alcohol is relatively rapidly metabolized by the liver and its effects can only be assessed if the alcohol levels are measured soon after the presentation and correlated with the ECG findings. Our review provides a practical guide to recognize certain ECG changes and attribute them to acute alcohol intoxication rather than labelling them as idiopathic or non-specific. Serial ECGs should be done during hospitalization as most changes will resolve with the elimination of alcohol from the system. If these ECG alterations fail to resolve with the treatment of alcohol intoxication, an alternate cause should be considered.

Ethanol consumption might be associated with intra myocardial [11] as well as adrenal release of catecholamines [12], abnormal autonomic nervous system discharges or electrophysiological consequences of acetaldehyde (the metabolite of ethanol) [12]. Other likely causes of arrhythmias in the setting of acute alcohol intoxication are deranged plasma electrolytes, particularly low potassium and magnesium [13]. High serum alcohol levels may interfere with sodium, potassium and calcium ion channels in the heart [14]. Moreover, alcohol may also lead to instabilities in autonomic regulation of cardiac rhythm, thereby causing arrhythmias [15].

Alcohol intoxication can cause prolongation of the PR, QRS and QT-intervals and sensitize the myocardium to atrial arrhythmias as well as life threatening ventricular arrhythmias [16]. The sudden cessation of alcohol intake results in beta-adrenergic stimulation and increase in catecholamine levels. Thus, patients may be prone to several arrhythmias during alcohol detoxification [17]. ECG changes are observed when serum concentration of alcohol is >600 mg/100 mL *(130 mmol/L)* [18, 19]. A decrease in the rate of rise of phase “O” of the action potential and the amplitude of the action potential is observed with intoxication [18, 20].

Bradycardia and atrioventricular (AV) block have been occasionally reported with acute alcohol intoxication. It is possible that decreased calcium as well as sodium currents are related with AV block after alcohol consumption [13]. Van Stigt *et al*. [13] studied 8 cases of AV block following acute alcohol consumption. Five patients had first-degree AV block and 3 patients presented with second-degree AV block. Of the 5 patients with first-degree AV block, 1 evolved into a third-degree AV block. The authors did not identify an apparent dose-response relationship between serum alcohol concentration and degree of AV block. In 7 patients, complete recovery occurred, while in 1 patient first-degree AV block persisted during follow-up and may have been present previously [13].

Alcohol intoxication has been reported to cause atrial fibrillation [21, 22]. It is not clear whether P-wave prolongation with alcohol intoxication is related to atrial fibrillation. As reported in the AFFIRM study [23], P wave duration > 135 m sec in lead II was a risk factor for atrial fibrillation recurrence after cardioversion. The typical presentation, usually described as “holiday heart syndrome” is characterized as an acute conduction impairment associated with heavy (>600 mg/100 ml or 130 mmol/L) ethanol consumption in a person without any underlying cardiac disorder and normalization of the rhythm with avoidance of alcohol [1]. Aaesebo *et al*. [3] reported that 1 out of 84 patients in their study developed transitory atrial fibrillation approximately 12 h after admission. Priest *et al*. [10] also reported cases of atrial fibrillation among the 20 abnormal ECGs evaluated by them but did not report a specific number for this. We speculate that atrial fibrillation with acute alcohol intoxication may be more common than what is reported in the literature but transient in nature, so not necessarily detected by a single ECG. It is more commonly associated with chronic alcohol consumption which can lead to dilated cardiomyopathy, hence making the heart prone to atrial fibrillation.

Oda *et al*. [24] reported that alcohol-induced coronary vasospasm may continue for up to 9 h after intake, even if the plasma concentration of ethanol returns to normal. They suggested that low concentrations of prostaglandin after alcohol consumption may be responsible for this effect. Large quantities (1.5 g/kg) of alcohol cause thromboxane mediated activation of platelets, trigger the inhibitors of plasminogen [25] and have an acute inhibitory effect on fibrinolytic action [26]. Bylik *et al*. [27] reported a 19 year old patient with acute ST elevation myocardial infarction despite normal coronary arteriography. The etiology was thought to be acute alcohol intoxication.

Ventricular arrhythmias are clinically important in alcoholics and may lead to sudden cardiac death. Achaiah *et al*. [28] reported a 56 year old man who had a cardiac arrest due to ventricular fibrillation while sleeping. He had consumed 1 bottle of wine before sleeping. After resuscitation, his ECG revealed an intermittent type 1 Brugada configuration, so the ventricular fibrillation was possibly caused by acute effect of alcohol on the already arrhythmogenic heart.

It is important to note that the prognosis of arrhythmias due to alcohol intoxication is good and recovery usually follows the disappearance of alcohol from the blood.

## CONCLUSION

The most common electrocardiogram changes associated with acute alcohol intoxication are (in decreasing order of frequency) P-wave and QTc prolongation, followed by T-wave abnormalities and QRS prolongation. Recovery is almost always complete after elimination of alcohol from the system. Further studies are required to better establish the association of acute alcohol intoxication with ECG abnormalities.

## Figures and Tables

**Table 1:** The included studies and their characteristics.

**S. No**	**Author**	**Type of study**	**Year of Publication**	**No of subjects**	**Age (Years)**	**Sex**	**Serum Ethanol level**
1	Cardy^9^	Case control	1996	10	23-27	6-Female, 4-Male	25-75 mg/dl
2	Priest^10^	Case control	1996	37	Not reported	Not reported	Unknown
3	Uyarel^8^	Crossover study	2005	10	25-33	Male	85-134 mg/dl
4	Aasebo^3^	Case control	2007	84	39 (mean)	52-Male,32-Female	367 mg/dl

**Table 2:** Electrocardiogram changes reported in the included studies.

**S. No**	**Author**	**Type of study**	**No of subjects**	** Results**					
				P-wave prolongation	QRS Prolongation	QTc Prolongation	ST-segment depressions	T-Wave abnormalities	Atrial Fibrillation
1	Cardy^9^	Case control	10	9	10				
2	Priest^10^	Case control	37				3	19	
3	Uyarel^8^	Cross control	10	10					
4	Aasebo^3^	Case control	84	71		80			1
**Total**			**141**	**90**	**10**	**80**	**3**	**19**	**1**
%				63.8	7	56	2.12	13.47	0.7
									

## References

[1] Ettinger P.O., Wu C.F., De La Cruz C., Weisse A.B., Ahmed S.S., Regan T.J. (1978). Arrhythmias and the “Holiday Heart”: Alcohol-associated cardiac rhythm disorders.. Am. Heart J..

[2] George A., Figueredo V.M. (2010). Alcohol and arrhythmias: A comprehensive review.. J. Cardiovasc. Med. (Hagerstown).

[3] Aasebø W., Erikssen J., Jonsbu J., Stavem K., Stavem K. (2007). ECG changes in patients with acute ethanol intoxication.. Scand. Cardiovasc. J..

[4] Djoussé L., Levy D., Benjamin E.J., Blease S.J., Russ A., Larson M.G., Massaro J.M., D’Agostino R.B., Wolf P.A., Ellison R.C. (2004). Long-term alcohol consumption and the risk of atrial fibrillation in the Framingham Study.. Am. J. Cardiol..

[5] Kupari M., Koskinen P., Suokas A. (1991). Left ventricular size, mass and function in relation to the duration and quantity of heavy drinking in alcoholics.. Am. J. Cardiol..

[6] Engler R., Ray R., Higgins C.B., McNally C., Buxton W.H., Bhargava V., Shabetai R. (1982). Clinical assessment and follow-up of functional capacity in patients with chronic congestive cardiomyopathy.. Am. J. Cardiol..

[7] Marriott H.J. (1964). Electrocardiographic abnormalities, conduction disorders and arrhythmias in primary myocardial disease.. Prog. Cardiovasc. Dis..

[8] Uyarel H, Ozdol C, Karabulut A, Okmen E, Cam N (2005). Acute alcohol intake and P-wave dispersion in healthy men.. Anadolu Kardiyoloji Dergisi.

[9] Cardy M.A., Donnerstein R.L., Kelly L.F., Bittner N.H., Palombo G.M., Goldberg S.J. (1996). Acute effects of ethanol ingestion on signal-averaged electrocardiograms.. Am. J. Cardiol..

[10] Priest R.G., Binns J.K., Kitchin A.H. (1966). Electrocardiogram in alcoholism and accompanying physical disease.. BMJ.

[11] James T.N., Bear E.S. (1967). Effects of ethanol and acetaldehyde on the heart.. Am. Heart J..

[12] Perman E.S. (1958). The effect of ethyl alcohol on the secretion from the adrenal medulla in man.. Acta Physiol. Scand..

[13] van Stigt A.H., Overduin R.J., Staats L.C., Loen V., van der Heyden M.A. (2016). A Heart too Drunk to Drive; AV Block following Acute Alcohol Intoxication.. Chin. J. Physiol..

[14] Klein G., Gardiwal A., Schaefer A., Panning B., Breitmeier D. (2007). Effect of ethanol on cardiac single sodium channel gating.. Forensic Sci. Int..

[15] Reed S.F., Porges S.W., Newlin D.B. (1999). Effect of alcohol on vagal regulation of cardiovascular function: Contributions of the polyvagal theory to the psychophysiology of alcohol.. Exp. Clin. Psychopharmacol..

[16] (1978). L, Reddy CV, Becker W, Oh KC, Kim SG Electrophysiological properties of alcohol in man.. J. Electrocardiol..

[17] Talbott G.D. (1975). Primary alcoholic heart disease.. Ann. N. Y. Acad. Sci..

[18] Wakim K.G. (1946). The effects of ethyl alcohol on the isolated heart.. Fed. Proc..

[19] Ghadri J.R., Templin C., Duru F., Lüscher T.F., Haegeli L.M. (2013). Holiday heart block: Alcohol-induced PR prolongation.. Am. J. Med..

[20] Eilam O., Heyman S.N. (1991). Wenckebach-type atrioventricular block in severe alcohol intoxication.. Ann. Emerg. Med..

[21] Thornton JR (1984). Atrial fibrillation in healthy non-alcoholic people after an alcoholic binge..

[22] Koskinen P., Kupari M., Leinonen H. (1990). Role of alcohol in recurrences of atrial fibrillation in persons less than 65 years of age.. Am. J. Cardiol..

[23] Raitt M.H., Volgman A.S., Zoble R.G., Charbonneau L., Padder F.A., O’Hara G.E., Kerr D. (2006). Prediction of the recurrence of atrial fibrillation after cardioversion in the Atrial Fibrillation Follow-up Investigation of Rhythm Management (AFFIRM) study.. Am. Heart J..

[24] Oda H., Suzuki M., Oniki T., Kishi Y., Numano F. (1994). Alcohol and coronary spasm.. Angiology.

[25] Numminen H., Syrjälä M., Benthin G., Kaste M., Hillbom M. (2000). The effect of acute ingestion of a large dose of alcohol on the hemostatic system and its circadian variation.. Stroke.

[26] van de Wiel A., van Golde P.M., Kraaijenhagen R.J., von dem Borne P.A., Bouma B.N., Hart H.C. (2001). Acute inhibitory effect of alcohol on fibrinolysis.. Eur. J. Clin. Invest..

[27] Biyik I., Ergene O. (2006). Acute myocardial infarction associated with heavy alcohol intake in an adolescent with normal coronary arteries.. Cardiol. Young.

[28] Achaiah A., Andrews N. (2016). Intoxication with alcohol: An underestimated trigger of Brugada syndrome?. JRSM Open.

